# Online HD-tRNS Over the Right Temporoparietal Junction Modulates Social Inference But Not Motor Coordination

**DOI:** 10.1523/ENEURO.0155-25.2025

**Published:** 2025-10-08

**Authors:** Quentin Moreau, Vincent Chamberland, Lisane Moses, Gabriela Milanova, Guillaume Dumas

**Affiliations:** ^1^CHU Sainte-Justine Azrieli Research Center, Montreal, Quebec H3T ICS, Canada; ^2^Department of Psychiatry and Addiction, University of Montreal, Montreal, Quebec H3T 1J4, Canada; ^3^Department of Psychology, University of Montreal, Montreal, Quebec H2V 259, Canada; ^4^Mila - Quebec AI Institute, Montreal, Quebec H2S 3H1, Canada

**Keywords:** high-definition transcranial random noise stimulation, human–machine interaction, right temporoparietal junction, self–other distinction, social inference, Turing test

## Abstract

Social interactions are fundamental to human cognition, with the right temporoparietal junction (rTPJ) playing a key role in integrating motor coordination and social inference. While transcranial random noise stimulation (tRNS) is a promising technique for modulating cortical excitability in real time, its effect on dynamic social processes remains largely unexplored. This study applied high-definition tRNS (HD-tRNS) over the rTPJ during an interactive task to modulate motor coordination and social inference. Eighty neurotypical adults (49 female) were equally distributed across two experiments: Experiment 1, a block design with randomized active and sham stimulation blocks; or Experiment 2, a trial-by-trial design with intermixed stimulation protocols. Participants performed a coordination task with a covert virtual partner programmed to behave cooperatively or competitively. Kinematic data and self-reported attributions of humanness and cooperativeness were analyzed. The results showed that HD-tRNS over the rTPJ did not affect motor coordination or overall task performance in either experiment. However, in Experiment 1, active stimulation progressively reduced attributed humanness and cooperativeness toward the competitive virtual partner, suggesting enhanced detection of antagonistic intent. This gradual modulation of social inference was absent in Experiment 2, where frequent protocol switching likely disrupted the buildup of stimulation effects. Together, these findings highlight the rTPJ's causal role in self–other distinction, underscore the importance of stimulation protocol design in shaping social cognition, and support the exploration of targeted neuromodulation in clinical and developmental populations with atypical social cognition.

## Significance Statement

Social interactions rely on our ability to infer others’ intentions, including distinguishing between cooperative and competitive behavior: a process involving the right temporoparietal junction (rTPJ). Here, we used high-definition transcranial random noise stimulation (HD-tRNS) to test the rTPJ's causal role during live social interactions with an adaptive virtual partner. While stimulation did not affect motor coordination, repeated application led participants to gradually attribute less humanness and cooperativeness to a covertly competitive partner, suggesting enhanced sensitivity to competitive intent. These findings provide new insights into the rTPJ's contribution to self–other distinction, demonstrate the potential of HD-tRNS to investigate and modulate social inference, and have implications for understanding and potentially addressing social difficulties in conditions such as autism and schizophrenia.

## Introduction

Social interactions are fundamental to human life, supporting adaptation to sociocultural environments and facilitating effective coordination with others (Laland et al., 2001; Tomasello and Rakoczy, 2003). Understanding the neural correlates of such interactions is therefore crucial for uncovering the mechanisms that support social cognition. Early studies in social neuroscience primarily relied on unidirectional paradigms, in which isolated participants passively observed social stimuli, such as faces, gestures, or other-directed actions, without engaging in reciprocal exchange (Hari and Kujala, 2009; Hari et al., 2015). To address this limitation, virtual partners (VPs)—computer-controlled avatars modeled on human behavior (Kelso et al., 2009)—have been developed to simulate dynamic and ecologically valid interpersonal scenarios (Dumas et al., 2018). When combined with neuroimaging techniques, these paradigms offer a promising avenue for investigating the neural underpinnings of real-time interaction under controlled experimental conditions (Fairhurst et al., 2013, 2014; Pfeiffer et al., 2014; Moreau et al., 2020, 2023; Formica and Brass, 2024).

**Figure 1.**
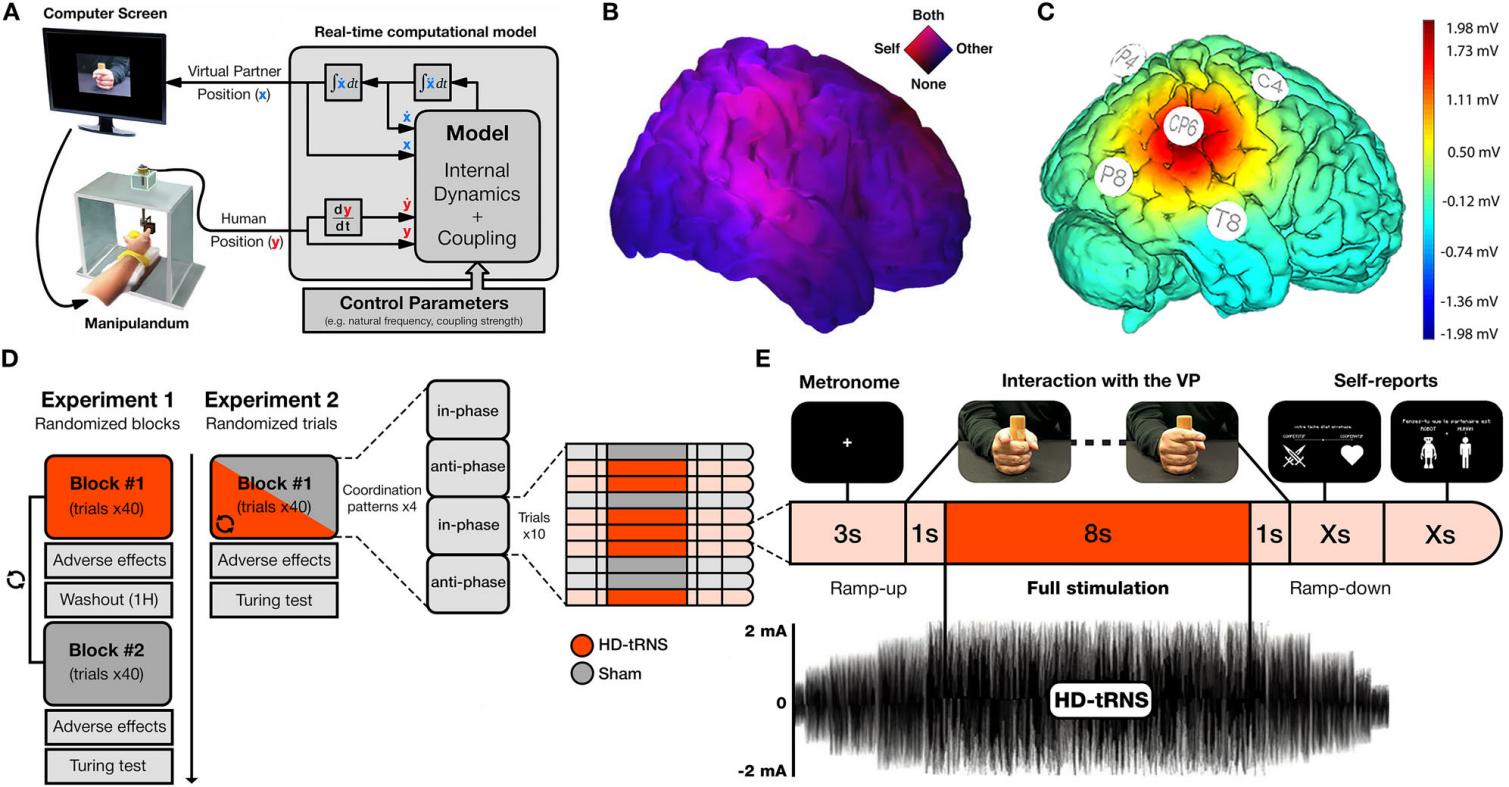
***A***, Schematic of the HDC paradigm. Real-time finger movements are captured and fed into a computational model of coordination dynamics, which integrates internal VP dynamics and its coupling with the participant's input to generate the VP's position onscreen. ***B***, Estimated cortical sources of high-density EEG revealing the rTPJ's involvement in the integration of self and other during the HDC paradigm, bridging low-level motor coordination with high-level social inference (adapted from Dumas et al., 2020). ***C***, Simulated cortical voltage distribution (in millivolts) induced by HD-tRNS targeting the rTPJ, based on finite element modeling in NIC 2.0 using the electrode montage from this study. ***D***, The two experimental designs: Experiment 1 with two randomized, alternating blocks of active stimulation and sham trials; Experiment 2 with a single block of randomized active stimulation and sham trials. ***E***, Example of a typical HDC trial with active stimulation.

Recently, the combination of high-density electroencephalography (EEG) with the Human Dynamic Clamp (HDC; Fig. 1*A*)—an interactive paradigm in which participants coordinate finger movements with a covert VP (Dumas et al., 2014; Kelso et al., 2014)—highlighted the right temporoparietal junction (rTPJ) as a critical cortical region for integrating “low-level” motor coordination with “high-level” social inference (Dumas et al., 2020; Fig. 1*B*). These findings align with extensive evidence recognizing the rTPJ as a key integrative hub in social cognition (Decety and Lamm, 2007; Lombardo et al., 2010; Bzdok et al., 2013; Carter and Huettel, 2013; Krall et al., 2015; Wu et al., 2015), mainly supporting functions related to the Theory of Mind (Saxe and Kanwisher, 2003; Schurz et al., 2017), self–other distinction (Decety and Sommerville, 2003; Sowden and Shah, 2014; Lamm et al., 2016; Quesque and Brass, 2019), and inhibition of motor imitation during social interaction (Brass et al., 2005, 2009; Spengler et al., 2009; Hogeveen et al., 2015; Sowden and Catmur, 2015).

Despite robust evidence from imaging and electrophysiology, correlational findings alone cannot establish a causal role of the rTPJ in social interaction. Noninvasive brain stimulation techniques, such as transcranial magnetic stimulation (TMS) and transcranial electrical stimulation (tES), enable researchers to transiently modulate cortical excitability and assess resulting behavioral changes (Miniussi et al., 2013; Cohen Kadosh, 2014; Polanía et al., 2018). Although the neurophysiological effects of brain stimulation remain highly variable and context dependent (López-Alonso et al., 2014; Willmot et al., 2024), warranting cautious interpretation, numerous studies suggest that neuromodulation of the rTPJ can impact a range of social cognitive processes (Donaldson et al., 2015; Ahmad et al., 2021). Notably, studies using high-frequency TMS or anodal transcranial direct current stimulation (tDCS) over the rTPJ have been reported to enhance self–other distinction (Santiesteban et al., 2012a, 2015), perspective-taking (Martin et al., 2020), intention attribution (Schuwerk et al., 2021; Panico et al., 2024), and increase prosocial and fair behavior in social decision-making tasks (Christian et al., 2023; Soutschek et al., 2024). In contrast, studies using low-frequency TMS or cathodal tDCS have been found to disrupt these functions (Mai et al., 2016; Bardi et al., 2017; Coll et al., 2017; Era et al., 2020). However, the predominant use of offline protocols has limited our understanding of how online brain stimulation might shape ongoing, dynamic social interactions.

Transcranial random noise stimulation (tRNS) is a relatively new tES technique that delivers random current fluctuations within a broad frequency band to modulate cortical excitability (Terney et al., 2008). While its neurophysiological mechanisms are still being investigated, tRNS is thought to operate via stochastic resonance, a process that enhances the detection of weak signals by increasing the signal-to-noise ratio in targeted brain regions (McDonnell and Abbott, 2009; Van Der Groen and Wenderoth, 2016; Pavan et al., 2019; Battaglini et al., 2023). Notably, short sessions of tRNS have been shown to induce acute changes in both neuronal activity and behavior (Potok et al., 2021, 2022), making it a promising tool for online brain stimulation in interactive paradigms (Cappelletti et al., 2013; Pirulli et al., 2013; Van Der Groen et al., 2022).

In this study, we tested whether tRNS over the rTPJ modulates participants’ motor coordination and social inference when interacting with the VP of the HDC task, consistent with the proposed integrative role of the rTPJ in bridging low- and high-level social cognitive processes. We conducted two complementary experiments: Experiment 1 assessed the acute effects of online tRNS across two randomized blocks, while Experiment 2 examined the consistency and specificity of these effects across a single block of randomized trials.

## Materials and Methods

### Participants

We recruited 80 French-speaking, right-handed participants with normal or corrected-to-normal vision, no prior history of neuropsychiatric conditions or movement disorders, and no contraindication to tES (Bikson et al., 2016; Antal et al., 2017). We assigned 40 participants to Experiment 1 (24 female; mean age, 29.6; SD = 11.7) and 40 to Experiment 2 (25 female; mean age, 32.6; SD = 13.5). One participant from Experiment 1 was excluded due to noncompliance with task instructions. All participants provided written informed consent and received monetary compensation of CAD $30. Experiment 1 lasted ∼3 h, while Experiment 2 lasted ∼2 h. The study protocol was approved by the Ethics Review Board of the CHU Sainte-Justine (September 19, 2022) and adhered to the ethical principles outlined in the Declaration of Helsinki.

### Behavioral task

The HDC paradigm (Dumas et al., 2014; Kelso et al., 2014) is a real-time interactive task designed to simulate dynamic social coordination (Fig. 1*A*). Seated ∼60 cm from a 27 inch monitor (2,560 × 1,440 pixels, 144 Hz), each participant rested their right forearm on a U-shaped support (21.5 × 8 × 4 cm) and grasped a vertical Plexiglas cylinder (4.5 × 3 cm), leaving only the index finger free to move. The hand was oriented in the sagittal plane, with the distal phalanx of the index finger inserted into the circular opening (2 cm in diameter) of a Plexiglas block mounted on a freely rotating metallic stem (18 cm in length). This apparatus, fixed atop a Plexiglas box (30.5 × 31.5 × 20 cm) positioned ∼50 cm to the participant's right, enabled nearly frictionless flexion-extension of the index finger in the horizontal plane about the metacarpophalangeal joint. Angular displacement was recorded in real time using a linear potentiometer, and the resulting kinematic data stream was continuously fed into the Haken–Kelso–Bunz model of coordination dynamics (Haken et al., 1985) to drive the finger movements of a VP displayed on the monitor.

Each trial began with a visual cue indicating the required coordination pattern, either in-phase (imitative) or anti-phase (complementary), according to the randomized condition. Participants then synchronized their finger movements to a steady 1.6 Hz auditory metronome for 3 s, after which the VP's finger appeared on the screen and began moving in response to the participant's behavior. Participants maintained their rhythmic oscillations throughout the interaction while attempting to achieve the instructed coordination pattern. The VP behaved either cooperatively, by adapting to support the participant's goal, or competitively, by opposing it.

Each trial yielded three quantitative measures derived from the recorded kinematic data: a motor score, quantifying the morphological resemblance between the participant's and the VP's movement amplitudes; a coordination score, reflecting the degree of temporal phase alignment between their oscillatory movements; and a task score, indicating how closely the participant's behavior matched the instructed coordination pattern. At the end of each trial, participants provided two self-report scores: a cooperativeness score, evaluating the participant's ability to attribute intention toward the VP's behavior as either “cooperative” or “competitive,” and a humanness score, based on a binary rating indicating whether they believed they had interacted with a human or a computer. Detailed descriptions of the HDC scores are available in Baillin et al. (2020).

### HD-tRNS montage

A Starstim8 tES device (Neuroelectrics) was used to deliver high-definition tRNS (HD-tRNS) through five Ag/AgCl electrodes (1 cm radius, π cm² contact area) arranged in a 4 × 1 ring configuration on a neoprene head cap, according to the international 10–10 system. To target the rTPJ, the central electrode was placed at CP6, consistent with montages adopted in previous tDCS studies (Santiesteban et al., 2012b, 2015; Sowden et al., 2015; Vandenbroucke et al., 2016; Esse Wilson et al., 2018; Martin et al., 2020; Wu et al., 2023; Cesari et al., 2024). The central electrode served as the active site and delivered the full stimulation current, while the four surrounding return electrodes (C4, T8, P4, P8) each received 25% of the returning current (Fig. 1*C*).

The applied current was ∼2 mA (1,935 µA, peak-to-baseline), with no DC offset, sampled at 1,280 Hz, and had a standard deviation of 645 µA. The signal was drawn from the full spectrum of the available high-frequency range (100–500 Hz) to maximize cortical excitability (Moret et al., 2019). A conductive gel was applied to all electrodes to ensure optimal contact and minimize impedance, which was verified prior to stimulation using the built-in impedance check of Neuroelectrics’ Instrument Controller (NIC 2.0). Stimulation was initiated only when all electrodes registered below 10 kΩ.

### Procedure and experimental design

Upon arrival, participants were seated and fitted with the HD-tRNS headset. They received standardized instructions regarding the HDC task and were told that some trials might involve interacting with a human partner located in another room, while others could feature a virtual agent designed to simulate human behavior. All sessions were administered by the same experimenter to reduce variability in instruction delivery.

In Experiment 1 (Fig. 1*D*), participants completed two blocks of 40 trials, with each block corresponding to either the active or the sham stimulation condition. Block order was counterbalanced across participants. Within each block, the VP's behavior (cooperative or competitive) and the required coordination pattern (in-phase or anti-phase) were pseudorandomized to balance trial counts across conditions while minimizing potential confounds related to task exposure. A 1 h washout period separated the two blocks to ensure recovery from stimulation, as effects from similar 2 mA tRNS protocols lasting ∼10 min typically subside within 40 min (Laczó et al., 2014).

Active HD-tRNS trials consisted of a 4 s ramp-up, 8 s of full stimulation during interaction with the VP, and a 4 s ramp-down, totaling 16 s (Fig. 1*E*). In contrast, sham trials mimicked the sensory experience of stimulation by including only the ramp-up and ramp-down phases, without delivering sustained current. Each active trial delivered ∼7.7 millicoulombs (mC) of current to the scalp, while each sham trial delivered ∼4.8 mC. Across all active trials in a block, this resulted in 5.33 min of full stimulation (10.67 min including ramp phases) and a cumulative dose of ∼308 mC. Sham blocks delivered 5.33 min of ramp-only stimulation, with an estimated cumulative dose of ∼192 mC.

Experiment 2 (Fig. 1*D*) followed a similar overall procedure but differed in design. Participants completed a single block of 40 trials in which the stimulation protocol, VP behavior, and coordination pattern were all pseudorandomized on a trial-by-trial basis, ensuring that no identical combination of conditions occurred consecutively. Since Experiment 2 included only 20 active and 20 sham trials, the total duration of full stimulation amounted to 2.67 min (5.33 min including ramp phases), delivering a cumulative dose of ∼154 mC. Sham trials contributed an additional 2.67 min of ramp-only stimulation, with an estimated cumulative dose of ∼96 mC, resulting in a total charge of ∼250 mC across the block.

After each block in Experiment 1, and after the single block in Experiment 2, participants completed a questionnaire to assess stimulation-related adverse effects. Upon completing the task, participants were debriefed and informed that all partners had been virtual. Immediately afterward, they completed a Turing-like assessment, indicating on a continuous scale (0–100%) the extent to which they believed they had interacted with a real human partner.

### Data processing

Using Python (v3.12.3), we imported the participants’ and VPs’ raw kinematic data (positions and velocities) and corrected outliers in the participants’ movement trajectories via a DBSCAN clustering algorithm (scikit-learn v1.6.0; Pedregosa et al., 2011). We then mean-centered the cleaned data and applied a second-order, double-pass Butterworth filter with a 20 Hz cutoff (scipy v1.14.1; Virtanen et al., 2020) to reduce high-frequency noise. Finally, we computed the continuous relative phase between the participants’ and the VPs’ movements using a Hilbert transform to estimate the phase and amplitude required for coordination, motor, and task scores. Humanness and cooperativeness scores were directly retrieved from the trial reports using participants’ self-assessments.

### Statistical analyses

In both experiments, we assessed the effect of HD-tRNS on all HDC scores using generalized linear mixed models (GLMMs) implemented in R (v4.3.3) via the *glmmTMB* package (v1.1.10; Brooks et al., 2017). For coordination, motor, task, and cooperativeness scores (bounded 0–1), we applied Beta regression with a logit link to account for their skewness and non-Gaussian distribution; for humanness scores (binary 0 or 1), we used a binomial GLMM with a logit link. The stimulation protocol (active/sham) and VP behavior (cooperative/competitive) were included as fixed effects, with random intercepts for participants to capture individual baseline differences. Trial number (*z*-scored) was included as a continuous fixed effect to control for potential cumulative stimulation or learning. Fixed effects were tested via Wald *z*-tests, and estimated marginal trends across trials were obtained using *emtrends* from the *emmeans* package (v1.10.5; Lenth, 2025). Pairwise contrasts among those trends (e.g., the effect of active stimulation vs sham within each VP behavior) were Bonferroni adjusted to account for multiple comparisons, and exponentiated coefficients (odds ratios) with 95% confidence intervals are reported.

Stimulation-related adverse effects reported by the 39 participants in Experiment 1 were analyzed using a multivariate analysis of variance (MANOVA) across all criteria (itching, pain, burning, heat, metallic taste, and fatigue) to assess blinding of the stimulation protocol.

### Code accessibility

All scripts used for data processing, statistical analyses, and experiment control are available on our laboratory's GitHub repository (https://github.com/ppsp-team/moreau2025). This includes the modified HDC code integrated with a custom MATLAB script that interfaces with Neuroelectrics’ NIC 2.0 software to automatically load and launch the active or sham stimulation protocol at the onset of each HDC trial.

## Results

### Experimental control measures

#### Belief in a human partner

Consistent with prior HDC studies, participants largely believed that another human took part in the experiment as an interacting partner: 72.22% (SD = 24.76; median = 80.00) in Experiment 1 and 69.05% (SD = 28.67; median = 77.50) in Experiment 2.

#### Stimulation-related adverse effects

Adverse effects were generally mild and transient in both experiments, with no participants choosing to withdraw. In Experiment 1, several participants reported light sensations such as itch, pain, burning, heat, and fatigue. However, only a few cases of moderate fatigue (*n* = 3 active; *n* = 5 sham) and moderate heat (*n* = 1 active) were reported, with no other moderate or severe effects observed.

**Figure 2.**
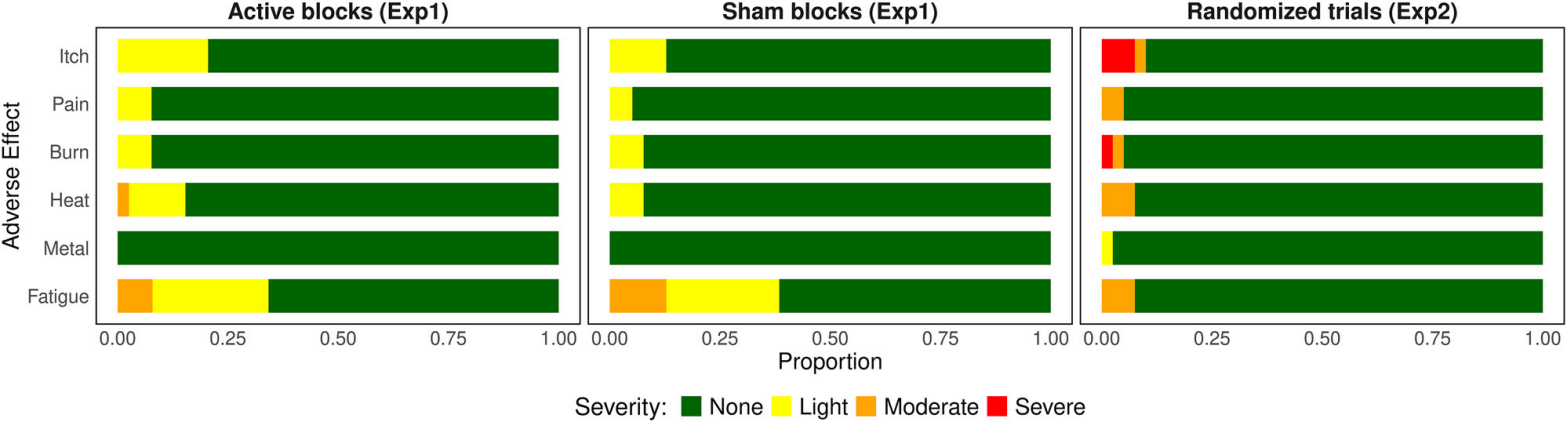
Proportional distribution of stimulation-related adverse effects by stimulation condition. Data reflect severity ratings for six adverse effects collected after each active and sham stimulation block (Experiment 1) or the single block of randomized trials (Experiment 2).

In Experiment 2, light effects were again reported across various categories, and some moderate and severe adverse effects occurred, including moderate itch (*n* = 1), severe itch (*n* = 3), moderate pain (*n* = 2), moderate or severe burning (*n* = 2), moderate heat (*n* = 3), moderate fatigue (*n* = 3), and one case of light metallic taste. Detailed proportions of all reported adverse effects are presented in Figure 2.

#### Blinding of the stimulation protocol

Specific to Experiment 1, the MANOVA results indicated no significant difference in adverse effects between the active and sham stimulation protocols (Wilks’ *λ* = 0.96, *F*_(5,72)_ = 0.67, *p* = 0.651). These findings suggest that, based on reported sensations, participants were unable to differentiate between the two stimulation protocols.

### Experiment 1: randomized block design

#### Low-level motor coordination

**Figure 3.**
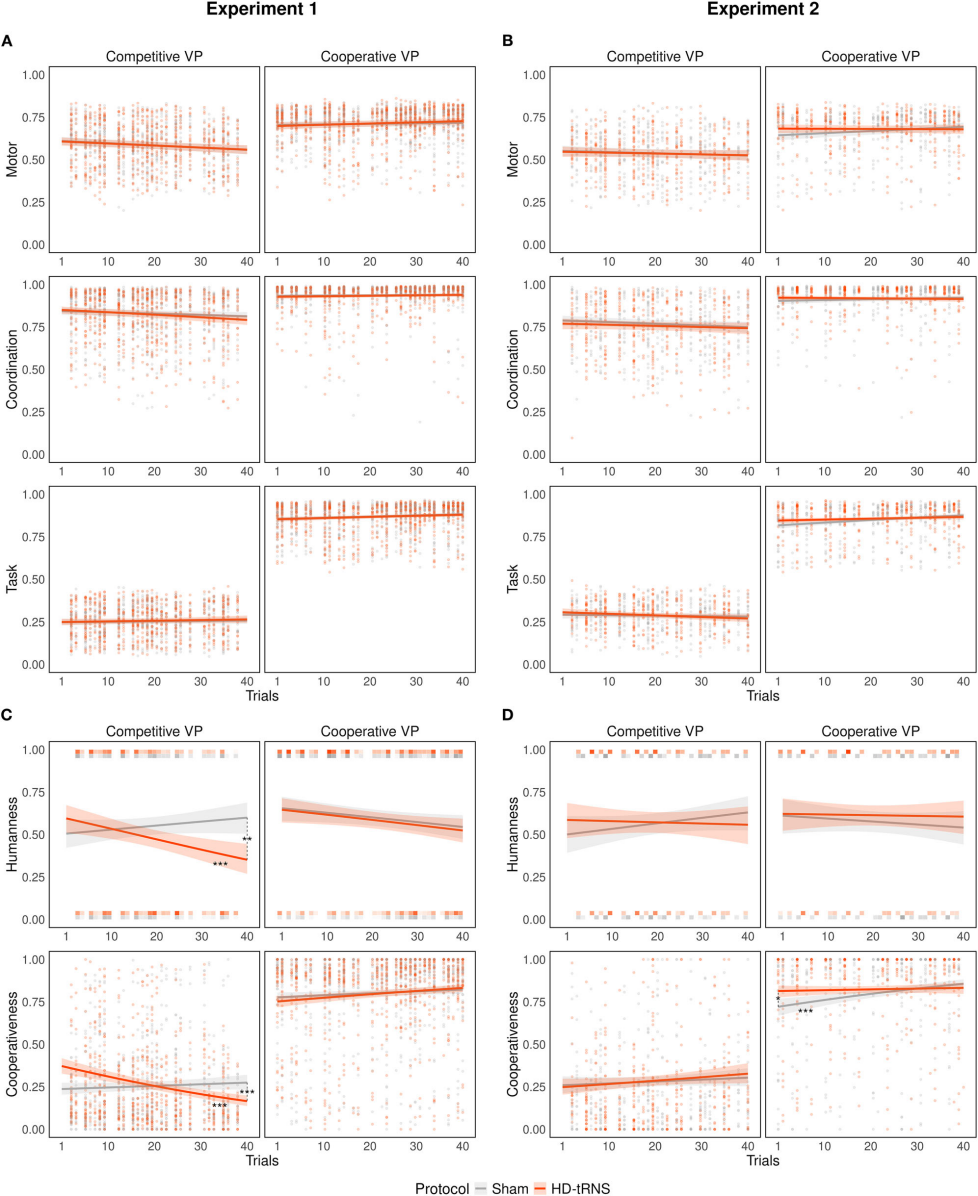
HDC scores across trials, stimulation protocols (sham vs HD-tRNS), and VP behavior (competitive vs cooperative), shown separately for Experiment 1 (left panels) and Experiment 2 (right panels). ***A***, ***B***, Low-level motor coordination scores predicted from the fitted GLMMs for Experiments 1 and 2, back-transformed from the logit scale to the original probability scale for visualization. ***C***, ***D***, High-level social inference scores predicted from GLMMs in Experiments 1 and 2. Colored shaded areas represent 95% confidence intervals around the model predictions. Data points represent individual trial-level observations. Asterisks denote significant post hoc contrasts or slopes (*p < 0.05, **p < 0.01, ***p < 0.001).

GLMM analyses revealed no significant main effect of HD-tRNS on coordination (Exp(β) = 0.96 [0.90, 1.02], *p* = 0.221), motor (Exp(β) = 1.00 [0.95, 1.04], *p* = 0.880), or task scores (Exp(β) = 1.02 [0.97, 1.08], *p* = 0.483), and no significant interactions were observed (Fig. 3*A*).

#### High-level social inference

In contrast, HD-tRNS significantly reduced humanness scores (Exp(β) = 0.72 [0.58, 0.90], *p* = 0.004), corresponding to a 28% decrease in the odds of attributing humanness to the VP under active stimulation. A significant three-way interaction was also observed between stimulation protocol, VP behavior, and trial progression (Exp(β) = 1.48 [1.09, 2.02], *p* = 0.012). Post hoc trend analysis revealed that, when interacting with a competitive VP, only active stimulation led to a significant decline in humanness scores over time (Exp(β) = 0.74 [0.62, 0.89], *p* = 0.001), with a slope significantly different from sham (Exp(β) = 1.50 [1.17, 1.93], *p* = 0.002; Fig. 3*C*). Conversely, when the VP was cooperative, no significant difference between protocols was observed (Exp(β) = 1.01, [0.84, 1.22], *p* = 1.000).

Further exploratory analysis revealed a significant interaction between stimulation protocols and baseline humanness scores (Exp(β) = 1.78 [1.22, 2.60], *p* = 0.003), suggesting that the effect of HD-tRNS diminished as baseline humanness scores increased. At low baseline levels (logit = −0.681, 10^th^ percentile), HD-tRNS reduced humanness scores by 42% compared with sham (Exp(β) = 0.58 [0.43, 0.78], *p* < 0.001). At high baseline levels (logit = 0.448, 90^th^ percentile), no significant difference was observed (Exp(β) = 1.11 [0.89, 1.39], *p* = 0.693).

Cooperativeness scores showed no main effect of HD-tRNS (Exp(β) = 1.00 [0.88, 1.13], *p* = 0.996). However, a significant three-way interaction was observed between stimulation protocol, VP behavior, and trial progression (Exp(β) = 1.56 [1.31, 1.84], *p* < 0.001). Post hoc trend analysis further showed that, during interactions with a competitive VP, active stimulation led to a significant decrease of cooperativeness scores over time (Exp(β) = 0.72 [0.66, 0.80], *p* < 0.001), with a slope significantly different from sham (Exp(β) = 1.47 [0.25, 0.52], *p* < 0.001). In contrast, no significant difference was found between protocols during cooperative VP interactions (Exp(β) = 0.94 [0.85, 1.04], *p* = 0.462; Fig. 3*C*).

### Experiment 2: randomized trial design

#### Low-level motor coordination

Consistent with Experiment 1, HD-tRNS had no significant main effect on coordination (Exp(β) =0.93 [0.85, 1.02], *p* = 0.141), motor (Exp(β) = 0.99 [0.93, 1.06], *p* = 0.833), or task scores (Exp(β) = 1.01 [0.95, 1.08], *p* = 0.785), and no significant interactions were observed (Fig. 3*B*).

#### High-level social inference

Humanness scores were also unaffected by HD-tRNS. No main effect was found (Exp(β) = 1.02 [0.77, 1.36], *p* = 0.884), and no significant interactions emerged, contrasting the significant effects observed in Experiment 1 (Fig. 3*D*).

Cooperativeness scores did not show a significant main effect of HD-tRNS (Exp(β) = 1.02 [0.86, 1.21], *p* = 0.780), but a three-way interaction again emerged between stimulation protocol, VP behavior, and trial progression (Exp(β) = 0.77 [0.61, 0.98], *p* = 0.034). However, post hoc trend analysis revealed that, unlike in Experiment 1, active stimulation had no significant effect on cooperativeness scores over time in the competitive VP condition (Exp(β) = 1.12 [0.98, 1.28], *p* = 0.084), and the contrast between stimulation protocols was not significant (Exp(β) = 0.95 [0.79, 1.14], *p* = 1.000).

Conversely, in the cooperative VP condition, only sham stimulation was associated with a significant upward trend in cooperativeness scores over time (Exp(β) = 1.28 [1.42, 1.44], *p* < 0.001), and a significant difference between stimulation protocols was observed (Exp(β) = 1.23 [1.05, 1.44], *p* = 0.018; Fig. 3*D*).

## Discussion

This study investigated the causal contribution of the rTPJ to real-time social interaction by applying HD-tRNS during the trials of the HDC paradigm. While stimulation did not affect low-level motor coordination, it selectively modulated higher-level social inference, specifically the attribution of humanness and cooperativeness during competitive interactions, but only under conditions that allowed sustained stimulation (Experiment 1). These findings highlight the potential for state-dependent and dose-sensitive neuromodulatory effects on social cognition.

### No effect of HD-tRNS on low-level motor coordination

Contrary to previous findings linking rTPJ modulation to motor coordination, we observed no effect of HD-tRNS on any coordination measures in either experiment. For instance, Era et al. (2020) reported that offline continuous theta burst TMS to the rTPJ selectively disrupted imitative behaviors during interaction with a VP. One explanation for this discrepancy may lie in task demands: while rTPJ can indirectly influence motor imitation-inhibition (Brass et al., 2005, 2009; Hogeveen et al., 2015; Sowden and Catmur, 2015), its role in simple in-phase or anti-phase coordination may be less critical. It is also possible that participants were already performing near the ceiling regarding motor skills, leaving limited room for behavioral modulation through stimulation. Additionally, it is worth considering that more prolonged or repeated stimulation may be necessary to produce measurable effects.

### HD-tRNS reduces humanness attribution during competitive interactions

In Experiment 1, participants progressively attributed less humanness to a covertly competitive VP under active stimulation. This effect shows that enhanced rTPJ excitability may facilitate the detection of antagonistic intent (Payne and Tsakiris, 2017; Filmer et al., 2019). Notably, the rTPJ is known to support mentalizing and agent recognition (Özdem et al., 2017; Ogawa and Kameda, 2019), and its inhibition has been shown to impair cognitive empathy (Mai et al., 2016; Coll et al., 2017). These findings also align with evidence that cooperative or synchronized interactions tend to increase perceived humanness (Koehne et al., 2016; Jastrzab et al., 2024), whereas adversarial dynamics diminish it (Zhang et al., 2016; Dumas et al., 2020).

Our exploratory analysis further revealed that the impact of the stimulation depended on participants’ baseline humanness attribution: active stimulation significantly reduced ratings among those with initially low scores but had little effect on participants who already attributed high humanness to the VP. This baseline-dependent modulation may reflect a ceiling effect, limiting further increases among participants already perceiving the VP as highly human, or a form of belief perseverance, whereby strong prior impressions resist change. Together, these results are consistent with predictive models of social inference in which the rTPJ integrates external cues (e.g., behavioral antagonism) with internally generated social expectations (Lamm et al., 2007; Carter and Huettel, 2013).

### HD-tRNS enhances recognition of competitive intent

In Experiment 1, a similar effect of active stimulation was observed on cooperativeness scores, with participants interacting with a covertly competitive VP becoming increasingly accurate in detecting its antagonistic behavior. This aligns with evidence that rTPJ supports perspective-taking abilities (Zhang et al., 2019; Martin et al., 2020) and control of self–other representations (Santiesteban et al., 2012b, 2015), while inhibitory stimulation of rTPJ has been shown to disrupt these functions (Tsakiris et al., 2008; Wang et al., 2016; Era et al., 2020). It has also been proposed that the rTPJ dynamically allocates attention to socially salient cues, particularly in ambiguous or competitive contexts where others’ intentions must be inferred (Bitsch et al., 2018). By enhancing this attentional mechanism, increased rTPJ excitability may support more precise differentiation between self- and other-generated actions and goals, which is especially critical when anticipating an adversary's behavior (Giardina et al., 2011).

However, this pattern did not replicate in Experiment 2. Despite a significant three-way interaction, post hoc analyses revealed no evidence of a stimulation effect in the competitive VP condition. This discrepancy, also seen with humanness scores, may result from the trial-level randomization design of Experiment 2, which might have disrupted the cumulative stimulation effects observed in the blocked design of Experiment 1.

Interestingly, another asymmetry emerged in the cooperative VP condition of Experiment 2: only sham stimulation trials led to a significant increase in cooperativeness attribution over time. One explanation is that HD-tRNS may have disrupted a learning effect present under sham or contributed to a ceiling effect by causing early saturation in cooperativeness scores. However, the absence of such a ceiling in Experiment 1 suggests that the results of Experiment 2 may reflect more nuanced interactions between stimulation, task adaptation, and attentional dynamics, underscoring the need for cautious interpretation and systematic replication.

### Cumulative stimulation is required to modulate high-level social inference

The differences between Experiments 1 and 2 suggest that the effects of HD-tRNS on social inference depend on the temporal structure of stimulation. Only the blocked design in Experiment 1, which allowed for sustained exposure to active stimulation, produced reliable reductions in attributed humanness and cooperativeness. In contrast, the trial-level randomization used in Experiment 2 may have disrupted the accumulation of neuromodulatory effects, preventing a measurable behavioral impact.

Although Experiment 1 was initially designed to probe the acute effects of online stimulation, the gradual emergence of behavioral changes across trials suggests that even short blocks may rely on a brief buildup of stimulation-induced plasticity. These findings underscore the importance of distinguishing between truly acute, trial-by-trial effects, and those requiring minimal but cumulative exposure, an important consideration when interpreting the null effects observed in Experiment 2.

This interpretation aligns with growing evidence that high-level social cognitive processes are more sensitive to repeated or sustained stimulation than to brief pulses. While short tRNS bursts can rapidly modulate excitability in perceptual and motor domains (Van Der Groen and Wenderoth, 2016; Van Der Groen et al., 2019; Potok et al., 2021, 2022), observable behavioral effects typically require 4–7 min of continuous offline stimulation (Chaieb et al., 2009, 2011; Haeckert et al., 2020). Notably, the only prior study applying tRNS to the rTPJ found that behavioral effects in a temporal attention task emerged gradually and peaked ∼15 min into continuous stimulation (Tyler et al., 2018).

### Limitations

Several limitations should be acknowledged. First, although we used a high-definition tRNS montage to target the rTPJ, the region encompasses multiple cytoarchitectonic subareas (e.g., the angular and supramarginal gyri; Bzdok et al., 2013; Krall et al., 2015; Doricchi, 2022). Consequently, the current spread may have affected neighboring regions, reducing anatomical specificity. Second, although participants were blinded to the stimulation protocol, the experimenter was not, which may have introduced subtle bias despite the use of standardized procedures. Third, since the Turing-like belief rating was administered after participants were informed that no human was involved, their responses may have been influenced by this disclosure. Fourth, we did not include a control stimulation site (e.g., the left TPJ, which has also been implicated in social inference and mentalizing; Ogawa and Kameda, 2019; Golec-Staśkiewicz et al., 2022; Hao et al., 2022), limiting our ability to attribute effects solely to the rTPJ. Finally, we did not assess individual responses to tRNS before the experiment, which may have introduced inter- and intra-individual variability (López-Alonso et al., 2014) that could have influenced stimulation efficacy and contributed to the variability in our results.

### Future directions

Building on these findings, future research could explore adaptive or closed-loop stimulation protocols that dynamically modulate neuromodulatory input based on real-time behavioral signals (Ramot and Martin, 2022). In addition, multi-site stimulation approaches may better engage the distributed frontoparietal networks involved in social interactions, potentially enhancing effects beyond those observed with focal rTPJ targeting (Dumas et al., 2020). Another promising direction is multibrain stimulation, in which the rTPJs of two interacting individuals are simultaneously targeted to examine causal contributions to interbrain synchrony, extending concepts from hyperscanning to intervention (Novembre and Iannetti, 2021; Dumas, 2022). Investigating these approaches in clinical and developmental populations with atypical social cognition, such as individuals with autism spectrum disorder or schizophrenia, may inform precision neuromodulation strategies (Medaglia et al., 2020) while deepening our understanding of the rTPJ's causal role in social interaction.

### Conclusion

Our findings provide novel evidence that short, online HD-tRNS sessions over the rTPJ do not influence basic motor coordination but can modulate high-level social inference, particularly in competitive contexts. These effects emerged cumulatively over repeated trials within the blocked design of Experiment 1, suggesting that sustained exposure may be necessary for neuromodulatory changes to accumulate. Together, the observed shifts in how participants judged the VP's humanness and cooperativeness highlight the rTPJ's causal role in integrating self–other representations during dynamic social interaction.

## Synthesis

Reviewing Editor: Alexander Soutschek, Ludwig-Maximilians-Universitat Munchen Decisions are customarily a result of the Reviewing Editor and the peer reviewers coming together and discussing their recommendations until a consensus is reached. When revisions are invited, a fact-based synthesis statement explaining their decision and outlining what is needed to prepare a revision will be listed below. The following reviewer(s) agreed to reveal their identity: Weronika Potok-Szybinska.

This manuscript presents a novel methodological approach to a relevant and timely research question, leveraging large sample sizes and a carefully designed experimental paradigm. The study is commendable for its innovative integration of non-invasive brain stimulation with interactive behavioral tasks. The authors present good practice of reporting, including null findings, which is a valuable contribution to the field. Below, I provide several suggestions to improve the balance and clarity of the manuscript.

1. The conclusions would benefit from a more cautious tone, especially given the limited replication between experiments. For instance, in Experiment 1, the authors highlight that active stimulation reduced the perceived cooperativeness of the competitive virtual partner (VP). However, in Experiment 2, a similar modulation in perceived cooperativeness (here increase) was observed in the sham condition with the cooperative VP. This asymmetry in interpretation is not sufficiently addressed. Moreover, Figure 3 displays only the results from Experiment 1, which further emphasizes the findings from that experiment at the expense of the broader dataset. Including analogous data from Experiment 2 and addressing these results more evenly would help provide a more balanced interpretation.

2. It remains unclear whether the observed improvement in perceived cooperativeness reflects cumulative effects of stimulation or simply increased task exposure. I encourage the authors to consider this potential confound and, if possible, include analyses (for example including the condition order as covariate) or a discussion to address it.

3. Regarding possible order effects in Experiment 1, it would be helpful to know whether the condition participants started with (active vs. sham stimulation) influenced the outcomes. Please report how many participants began with each condition. This information is important to evaluate potential time-on-task effects.

4. Page 5: "At the end of the procedure, participants were informed that no human was involved and completed a Turing-like test, rating their belief (0-100%) in having interacted with a human." - The procedure surrounding the Turing-like test raises questions about possible bias. Participants were informed before the test that no human was involved in the interaction. This may have influenced their subsequent belief ratings. The authors should explain the rationale for this sequence and consider whether it could have affected the results. Also, it seems that the results of this test was not reported.

5. On page 13, the sentence "This interaction suggests that rTPJ stimulation may have a ceiling effect on humanness attribution, where participants who already perceive the partner as highly human show less room for further reductions" is somewhat counterintuitive. Rather than invoking a ceiling effect, it might be more appropriate to discuss this in terms of belief perseverance or cognitive dissonance-i.e., that participants may be less likely to revise strong initial impressions. A revised interpretation along these lines could make the finding more intuitive.

6. On page 7, please clarify the unit "mC." Does this refer to total charge in millicoulombs?

7. In the caption of Figure 2B, please indicate whether the figure represents the current delivered through the electrodes or a simulation of the effective electric field induced in cortical tissue.

8. In the main text, please add that detailed adverse effect reports for both stimulation conditions are available in the supplementary material. This will help readers locate this important information more easily.

9. Please report the method used to ensure adequate electrode impedance-e.g., whether gel or saline solution was used-and whether impedance was monitored throughout the sessions.

In summary, this is a carefully conducted and timely study that brings methodological innovation to the field. The authors' inclusion of null results and detailed reporting is commendable. Addressing the points above would further improve the clarity, balance, and interpretability of the findings.

Further comments by the editor:

In Experiment 1, participants performed blocks with 40 trials under sham and active stimulation, separated by a 1 h wash-out period. I would like to know how long exactly participants were stimulated during a block? What do we know about the duration of the after-effects of tRNS? If it remains possible that the after-effects were not completely washed-out at the start of the new block, this should be added as a further limitation to the discussion section.

Please justify why data were analyzed with beta regressions rather than ordinal GLMMs, as implemented by e.g. the ordinal package in R.

Stimulation and VP behavior were modelled as fixed effects, but given that it is recommended to maximize the random effects structure of GLMMs, I ask the authors to model these predictors also as random slopes (this should be possible because these variables vary on a within-subject level).

In the post-hoc analyses of cooperativeness scores, did the authors correct the p-values for multiple comparisons? Given the post-hoc nature of the analyses, it seems appropriate to apply Bonferroni-correction here (it anyway should not change the significance of the effects, given that the relevant p-values in Experiment 1 are <0.001).

In the discussion section, it is stated that Experiment 2 revealed no stimulation effects on cooperativeness ratings, but this does not seem fully correct given the significant three-way interaction (p=0.034).

According to the introduction, a limitation of previous TPJ stimulation studies is that they all relied on offline protocols, but two recent studies found effects of online tDCS over the TPJ on social cognition (Christian, Kapetaniou, &Soutschek, 2023, SCAN; Soutschek, Sahin, &Tobler, 2024, PNAS). It might be worth citing these papers here.

## Author Response

Reviewer 1 comments:

1. The conclusions would benefit from a more cautious tone, especially given the limited replication between experiments. For instance, in Experiment 1, the authors highlight that active stimulation reduced the perceived cooperativeness of the competitive virtual partner (VP). However, in Experiment 2, a similar modulation in perceived cooperativeness (here increase) was observed in the sham condition with the cooperative VP. This asymmetry in interpretation is not sufficiently addressed.

Moreover, Figure 3 displays only the results from Experiment 1, which further emphasizes the findings from that experiment at the expense of the broader dataset. Including analogous data from Experiment 2 and addressing these results more evenly would help provide a more balanced interpretation.

We thank the reviewer for this constructive comment and have revised our manuscript to better address the asymmetry between experiments. We are now explicitly discussing the lack of parallel effects, providing possible explanations for such differences, and highlighting the need for cautious interpretation. A dedicated paragraph was added in the "HD-tRNS enhances recognition of competitive intent" section of the Discussion: "Interestingly, another asymmetry emerged in the cooperative VP condition of Experiment 2: only sham stimulation trials led to a significant increase in cooperativeness attribution over time. One explanation is that HD-tRNS may have disrupted a learning effect present under sham or contributed to a ceiling effect by causing early saturation in cooperativeness scores. However, the absence of such a ceiling in Experiment 1 suggests that the results of Experiment 2 may reflect more nuanced interactions between stimulation, task adaptation, and attentional dynamics, underscoring the need for cautious interpretation and systematic replication." In addition, a new Figure 3 now includes results from both Experiment 1 and Experiment 2 across all outcome measures. This change ensures that the data are presented more comprehensively and that the interpretation is not disproportionately weighted toward one experiment.

2. It remains unclear whether the observed improvement in perceived cooperativeness reflects cumulative effects of stimulation or simply increased task exposure. I encourage the authors to consider this potential confound and, if possible, include analyses (for example including the condition order as covariate) or a discussion to address it.

We agree this is an important point, and it was an oversight not to clarify this in the submitted manuscript. To minimize potential order or learning effects, conditions within each block (e.g., [protocol:sham / coordination:in-phase / VP behavior:cooperative], [protocol:stim / coordination: in-phase / VP behavior:competitive], etc.) were pseudo-randomized. The order of sham and active stimulation blocks was also counterbalanced across participants. We have now added the following clarification to the "Procedure" section of the Materials and Methods: "Within each block [of Experiment 1], the VP's behavior (cooperative or competitive) and the required coordination pattern (in-phase or anti-phase) were pseudo-randomized to balance trial counts across conditions while minimizing potential confounds related to task exposure." "Experiment 2 followed a similar overall procedure but differed in design. Participants completed a single block of 40 trials in which the stimulation protocol, VP behavior, and coordination pattern were all pseudo-randomized on a trial-by-trial basis, ensuring that no identical combination of conditions occurred consecutively." We also explored having Condition Order as fixed and random effects in our mixed models. However, these models failed to converge, likely due to overparameterization given the number of condition combinations and our sample size. We therefore defaulted to the simpler model reported in the manuscript. Given the pseudo-randomization of conditions and counterbalancing of stimulation order, we believe this design is sufficient to control for task exposure or learning effects.

3. Regarding possible order effects in Experiment 1, it would be helpful to know whether the condition participants started with (active vs. sham stimulation) influenced the outcomes. Please report how many participants began with each condition. This information is important to evaluate potential time-on-task effects.

In Experiment 1, the order of stimulation blocks (active vs. sham) was counterbalanced across participants (20 started with active stimulation, and 20 with sham). We have added this information to the "Procedure" section of the Materials and Methods for clarity: "In Experiment 1, participants completed two blocks of 40 trials, with each block corresponding to either the active or the sham stimulation condition. Block order was counterbalanced across participants." In Experiment 2, task and stimulation conditions were intermixed within a single block. We ensured that half of the participants began the block with a trial assigned to the active condition, and the other half with a sham trial.

4. Page 5: "At the end of the procedure, participants were informed that no human was involved and completed a Turing-like test, rating their belief (0-100%) in having interacted with a human." - The procedure surrounding the Turing-like test raises questions about possible bias. Participants were informed before the test that no human was involved in the interaction. This may have influenced their subsequent belief ratings. The authors should explain the rationale for this sequence and consider whether it could have affected the results. Also, it seems that the results of this test were not reported.

We acknowledge that disclosing the deception prior to the belief rating may have introduced bias in the opposite direction. Despite this, the Turing-like belief ratings reported in the "Experimental control measures/Belief in a human partner" section of the Results indicate that participants largely believed another human was involved in both experiments: "Consistent with prior HDC studies, participants largely believed that another human took part in the experiment as an interacting partner: 72.22% (SD = 24.76; median = 80.00) in Experiment 1, and 69.05% (SD = 28.67; median = 77.50) in Experiment 2. " We also added a mention of this potential confound in our "Limitation" section of the Discussion: "Third, since the Turing-like belief rating was administered after participants were informed that no human was involved, their responses may have been influenced by this disclosure." 5. On page 13, the sentence "This interaction suggests that rTPJ stimulation may have a ceiling effect on humanness attribution, where participants who already perceive the partner as highly human show less room for further reductions" is somewhat counterintuitive. Rather than invoking a ceiling effect, it might be more appropriate to discuss this in terms of belief perseverance or cognitive dissonance-i.e., that participants may be less likely to revise strong initial impressions. A revised interpretation along these lines could make the finding more intuitive.

We agree that our original interpretation may have overlooked a more psychologically grounded explanation, like belief perseverance or cognitive dissonance. We have revised the relevant passage in the "HD-tRNS reduces humanness attribution during competitive interactions" section of the Discussion to reflect both possibilities: "This baseline-dependent modulation may reflect a ceiling effect, limiting further increases among participants already perceiving the VP as highly human, or a form of belief perseverance, whereby strong prior impressions resist change." 6. On page 7, please clarify the unit "mC." Does this refer to total charge in millicoulombs? Yes, "mC" does refer to the total electrical charge delivered to the scalp in millicoulombs during stimulation. This represents the cumulative load applied through the HD-tRNS electrodes over the course of each stimulation cycle.

We have updated the "Procedure and experimental design" section of the Materials and Methods for clarity: "Each active trial delivered approximately 7.7 millicoulombs (mC) of current to the scalp, while each sham trial delivered about 4.8 mC." 7. In the caption of Figure 2B, please indicate whether the figure represents the current delivered through the electrodes or a simulation of the effective electric field induced in cortical tissue.

We have created a new Figure 1C with a caption clarifying that it represents a simulation of the induced electrical field computed via finite element modeling: "C, Simulated cortical voltage distribution (in millivolts) induced by HD-tRNS targeting the rTPJ, based on finite element modeling in NIC 2.0 using the electrode montage from this study." 8. In the main text, please add that detailed adverse effect reports for both stimulation conditions are available in the supplementary material. This will help readers locate this important information more easily.

We decided to integrate detailed adverse effect reports for both stimulation conditions in the "Experimental control measures/Stimulation-related adverse effects" section of the Results: "Adverse effects were generally mild and transient in both experiments, with no participants choosing to withdraw. In Experiment 1, several participants reported light sensations such as itch, pain, burning, heat, and fatigue. However, only a few cases of moderate fatigue (n = 3 active; n = 5 sham) and moderate heat (n = 1 active) were reported, with no other moderate or severe effects observed.

In Experiment 2, light effects were again reported across various categories, and some moderate and severe adverse effects occurred, including moderate itch (n = 1), severe itch (n = 3), moderate pain (n = 2), moderate or severe burning (n = 2), moderate heat (n = 3), moderate fatigue (n = 3), and one case of light metallic taste. Detailed proportions of all reported adverse effects are presented in Fig. 2." Moreover, to facilitate visualization, we have also created a new Figure 2, which displays proportional plots of all effects for all experiments and stimulation conditions.

9. Please report the method used to ensure adequate electrode impedance-e.g., whether gel or saline solution was used, and whether impedance was monitored throughout the sessions.

We used conductive gel (SignaGel®) to ensure optimal contact between the electrodes and scalp. Impedance was measured before stimulation using the Neuroelectrics NIC 2.0 software's built-in impedance check, which applies a 300 µA test current and provides color-coded feedback: green for 0-10 kΩ, orange for 10-15 kΩ, and red for 15-20 kΩ.

In Experiment 1, impedance was checked before each of the first and third EEG recordings, which preceded the two stimulation blocks. In Experiment 2, impedance was checked once before the single stimulation block. In all cases, impedance was maintained below 10 kΩ prior to stimulation onset.

Importantly, the NIC 2.0 software continuously monitors impedance during stimulation and automatically aborts the protocol if it exceeds 20 kΩ, providing an additional safeguard to ensure proper recordings and stimulation delivery throughout the session.

We have added these details to the "HD-tRNS montage" section of the Materials and Methods: "A conductive gel was applied to all electrodes to ensure optimal contact and minimize impedance, which was verified prior to stimulation using the built-in impedance check of Neuroelectrics' Instrument Controller (NIC 2.0). Stimulation was initiated only when all electrodes registered below 10 kΩ." Editor's comments:

1. In Experiment 1, participants performed blocks with 40 trials under sham and active stimulation, separated by a 1 h wash-out period. I would like to know how long exactly participants were stimulated during a block? What do we know about the duration of the after-effects of tRNS? If it remains possible that the after-effects were not completely washed-out at the start of the new block, this should be added as a further limitation to the discussion section.

In Experiment 1, each active stimulation trial lasted 16 seconds-8 seconds of ramp-up/down and 8 seconds of steady stimulation-totaling 40 trials and 10.67 minutes of stimulation (or 5.33 minutes of steady-state stimulation alone). The cumulative dose for the active block was approximately 308 mC. By contrast, the sham block involved only ramp-up/down phases (8 seconds per trial), totaling 5.33 minutes and 192 mC. We have added these details to the "Procedure and experimental design" section of the Materials and Methods: "Each active trial delivered approximately 7.7 millicoulombs (mC) of current to the scalp, while each sham trial delivered about 4.8 mC. Across all active trials in a block, this resulted in 5.33 minutes of full stimulation (10.67 minutes including ramp phases) and a cumulative dose of ~308 mC. Sham blocks delivered 5.33 minutes of ramp-only stimulation, with an estimated cumulative dose of ~192 mC." Although research on the duration of tRNS after-effects remains limited, existing studies suggest that after-effects scale with both current intensity and total stimulation duration. For instance, Terney et al. (2008) reported motor cortex excitability changes lasting up to ~1 hour following 10 minutes of 1 mA tRNS. Laczó et al. (2014) observed ~40 minutes of after-effects after 10 minutes of 2 mA tRNS. Conversely, brief stimulations with 1 mA tRNS lasting 5 and 6 minutes have been shown to produce short-lasting effects of approximately 10 minutes (Chaieb et al., 2011). Given that our protocol used ~2 mA over ~5.3 minutes of steady stimulation, the expected carry-over is estimated to be ~20 minutes. Therefore, we are confident that any residual effects were fully resolved before the onset of the second block, and we did not consider it necessary to add a limitation on this point in the discussion. These details were also added to the "HD-tRNS montage" section of the Materials and Methods: "A one-hour washout period separated the two blocks to ensure recovery from stimulation, as effects from similar 2 mA tRNS protocols lasting ~10 minutes typically subside within 40 minutes (Laczó et al., 2014)." 2. Please justify why data were analyzed with beta regressions rather than ordinal GLMMs, as implemented by e.g. the ordinal package in R.

As described in the manuscript, the coordination, motor, task, and cooperativeness attribution scores are continuous and constrained within the (0, 1) interval. To reflect these properties, we selected beta regression with a logit link (via glmmTMB), which is well-suited for modeling proportion-like outcomes and capturing the skewness and asymmetry observed in our data-without requiring discretization. While ordinal GLMMs are appropriate for categorical ordinal outcomes, we treated our scale as continuous to preserve the granularity of participant ratings. To ensure the robustness of this modeling choice, we also tested alternative approaches and found that beta regression consistently provided a better fit, as confirmed by diagnostic checks using the DHARMa package [v0.4.7]. For the binary humanness attribution scores, we used a binomial GLMM with a logit link. We have updated the "Data and statistical analysis" section of the Materials and Methods: "For coordination, motor, task, and cooperativeness scores (bounded 0-1), we applied Beta regression with a logit link to account for their skewness and non-Gaussian distribution; ..." Distribution plots (not included in the manuscript) are also provided here to support these modeling decisions:

Experiment 1 Experiment 2 3. Stimulation and VP behavior were modelled as fixed effects, but given that it is recommended to maximize the random effects structure of GLMMs, I ask the authors to model these predictors also as random slopes (this should be possible because these variables vary on a within-subject level).

We agree with the reviewer's suggestion to maximize random-effects structures. Initially, we attempted to include random slopes for all within-subject predictors (Protocol, VP behavior, and Trial). However, several models failed to converge, likely due to model complexity relative to our sample size (40 participants per experiment) and the multiple comparisons involved.

To maintain consistency across all outcome measures (motor, coordination, task, cooperativeness, and humanness), we employed the most complex random-effects structure that reliably converged for all dependent variables: modeling random intercepts for each participant while treating the within-subject predictors as fixed effects.

4. In the post-hoc analyses of cooperativeness scores, did the authors correct the p-values for multiple comparisons? Given the post-hoc nature of the analyses, it seems appropriate to apply Bonferroni-correction here (it anyway should not change the significance of the effects, given that the relevant p-values in Experiment 1 are <0.001).

We have now applied Bonferroni correction to all post-hoc analyses involving significant effects. As anticipated, the correction did not change the significance of any results, including the key effects in Experiment 1 where the p-values remained well below the corrected threshold. We have revised the "Statistical analyses" section of the Materials and Methods: "Pairwise contrasts among those trends (e.g., the effect of active stimulation vs. sham within each VP behavior) were Bonferroni-adjusted to account for multiple comparisons, and exponentiated coefficients (odds ratios) with 95% confidence intervals are reported." 5. In the discussion section, it is stated that Experiment 2 revealed no stimulation effects on cooperativeness ratings, but this does not seem fully correct given the significant three-way interaction (p=0.034).

The editor is correct that a significant three-way interaction was observed among stimulation protocol, VP behavior, and trial progression in Experiment 2 (p = 0.034). In direct contrast to Experiment 1, post-hoc analyses did not reveal significant trend effects of active stimulation in the competitive VP condition (p = 0.084), nor a significant contrast between stimulation protocols (p = 1.000). However, a significant upward trend was observed in the sham condition during cooperative VP interactions (p < 0.001), and the contrast between active and sham protocols was also significant (p = 0.018).

Following the reviewer's first comment, we have revised the "HD-tRNS enhances recognition of competitive intent" section of the Discussion to reflect the nuance of these findings more accurately and created a new Figure 3 displaying results from both Experiment 1 and Experiment 2 across all outcome measures.

6. According to the introduction, a limitation of previous TPJ stimulation studies is that they all relied on offline protocols, but two recent studies found effects of online tDCS over the TPJ on social cognition (Christian, Kapetaniou, &Soutschek, 2023, SCAN; Soutschek, Sahin, &Tobler, 2024, PNAS). It might be worth citing these papers here.

We thank the reviewer for bringing these recent studies to our attention. We have added both citations to the Introduction: "Notably, studies using high-frequency TMS or anodal transcranial direct current stimulation (tDCS) over the rTPJ have been reported to enhance self-other distinction (Santiesteban et al., 2012a, 2015), perspective-taking (Martin et al., 2020), intention attribution (Schuwerk et al., 2021; Panico et al., 2024), and increase prosocial and fair behavior in social decision-making tasks (Christian et al., 2023; Soutschek et al., 2024)."
